# Extranodal Extension Is an Independent Prognostic Factor in Papillary Thyroid Cancer: A Propensity Score Matching Analysis

**DOI:** 10.3389/fendo.2021.759049

**Published:** 2021-11-03

**Authors:** Tian-han Zhou, Bei Lin, Fan Wu, Kai-ning Lu, Lin-lin Mao, Ling-qian Zhao, Ke-cheng Jiang, Yu Zhang, Wei-Jun Zheng, Ding-cun Luo

**Affiliations:** ^1^ The Fourth Clinical Medical College, Zhejiang Chinese Medical University, Hangzhou, China; ^2^ Department of Surgical Oncology, Affiliated Hangzhou First People’s Hospital, Zhejiang University School of Medicine, Hangzhou, China; ^3^ The School of Public Health, Zhejiang Chinese Medical University, Hangzhou, China

**Keywords:** extranodal extension, papillary thyroid carcinoma, prognosis, propensity score matching, lymph node metastasis, the real world

## Abstract

**Purpose:**

To investigate the prognostic significance of extranodal extension (ENE) in papillary thyroid cancer (PTC).

**Methods:**

Seven hundred forty-three PTC patients were enrolled in the study from January 2014 to December 2017. The patients were dichotomized according to the presence of ENE. Logistic analysis was used to compare differences between the two groups. Kaplan–Meier (K-M) curve and propensity score matching (PSM) analyses were used for recurrence-free survival (RFS) comparisons. Cox regression was performed to analyze the effects of ENE on RFS in PTC.

**Results:**

Thirty-four patients (4.58%) had ENE. Univariate analysis showed that age, tumor size, extrathyroidal extension, and nodal stage were associated with ENE. Further logistic regression analysis showed that age, extrathyroidal extension, and nodal stage remained statistically significant. Evaluation of K-M curves showed a statistically significant difference between the two groups before and after PSM. Cox regression showed that tumor size and ENE were independent risk factors for RFS.

**Conclusions:**

Age ≥55 years, extrathyroidal extension, and lateral cervical lymph node metastasis were identified as independent risk factors for ENE. ENE is an independent prognostic factor in PTC.

## Introduction

With the rapid development of medical technology, the demand for evidence-based clinical practice and decision-making has progressively increased. Recent studies using real-world data (RWD) are increasingly high profile and have received increasing attention in the medical field (1). Within the context of a rapidly rising incidence of thyroid tumors, a wealth of clinical data about papillary thyroid carcinoma (PTC) has been accumulated (2).

In previous studies on PTC, approximately 20%–80% of patients have cervical lymph node metastasis when diagnosed (3, 4). However, PTC has an indolent clinical course despite the high incidence of lymph node metastasis. The prognosis of lymph node metastasis in PTC patients remains controversial (5). When PTC develops lymph node metastasis, some metastatic cancers may break through the node capsule into neighboring tissue and metastasize to regional organs, which is called extranodal extension (ENE) (6). On the one hand, it has been reported that 14.5% of PTC patients with ENE may relapse, and ENE is considered to be an independent risk factor for recurrence and survival (7). On the other hand, some scholars have pointed out that invasive ENE does not affect the survival of PTC patients (8).

However, the above studies were all retrospective studies conducted under natural conditions, and confounding factors on the prognostic significance of ENE were not eliminated. Therefore, the purpose of this study was to evaluate the prognostic significance of ENE in PTC using the propensity score matching (PSM) method, which will provide more updated, comprehensive evidence for clinical decision-making.

## Materials and Methods

### Patients and Clinicopathological Data

**Figure 1:**
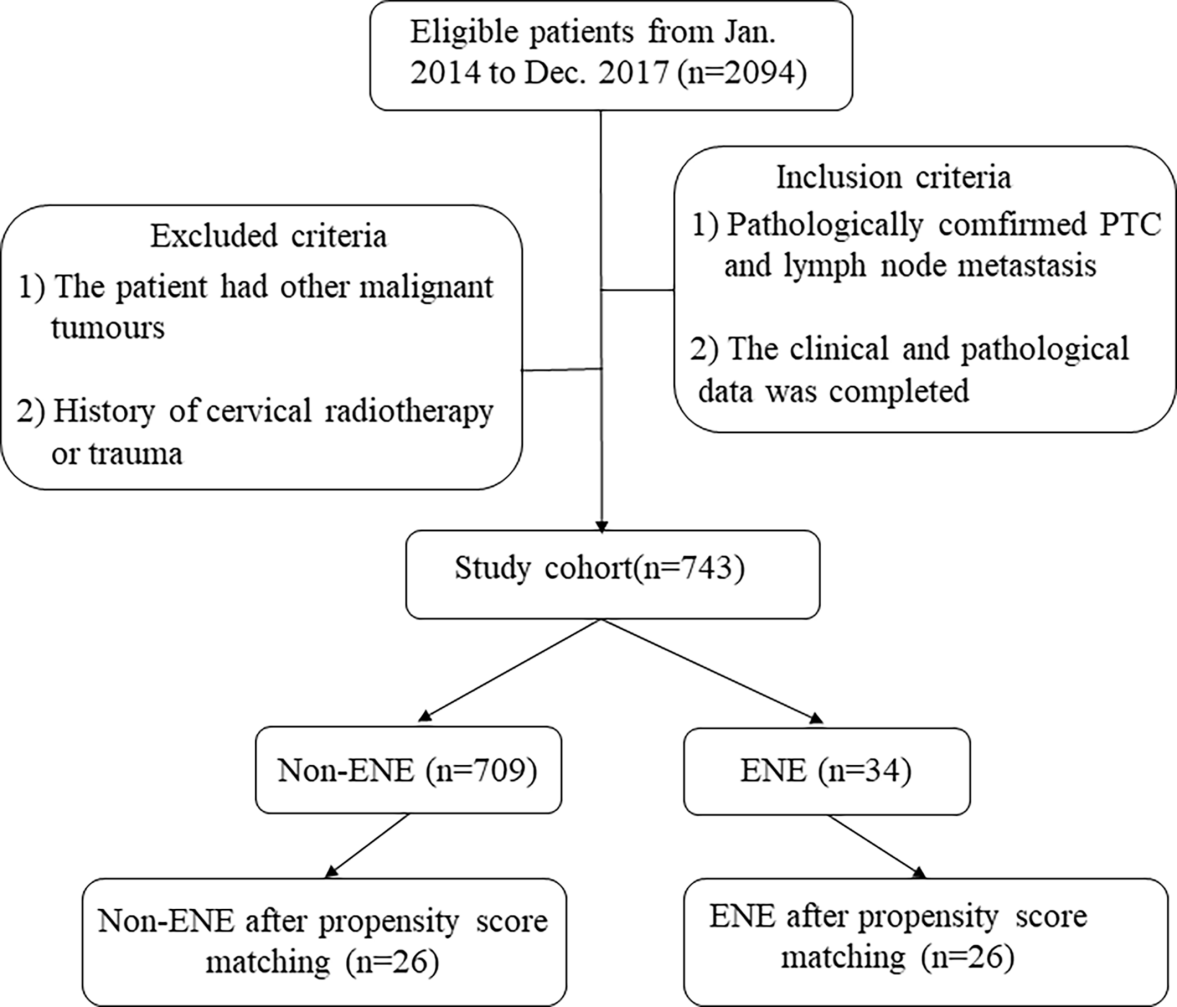
Process of patient enrollment into the study cohort and included patients. ENE, extranodal extension; PTC, papillary thyroid carcinoma.

We retrospectively reviewed the electronic medical records of 2,094 PTC patients in the Affiliated Hangzhou First People’s Hospital, Zhejiang University School of Medicine, from January 2014 to December 2017. Then, patients were selected according to the following inclusion criteria: 1) PTC and cervical lymph node metastasis were confirmed by pathologic examination and 2) the clinical and pathological data were complete. The exclusion criteria were as follows: 1) the patient had other malignant tumors and 2) the patient had a history of cervical radiotherapy or trauma. All the patients who underwent surgery signed informed consent forms, and all the study contents were approved. The process of patient enrollment to the study cohort is shown in **Figure 1**. The present study was approved by the ethics committee of our institution.

### Evaluation of Extranodal Extension and Extrathyroidal Extension

**Figure 2:**
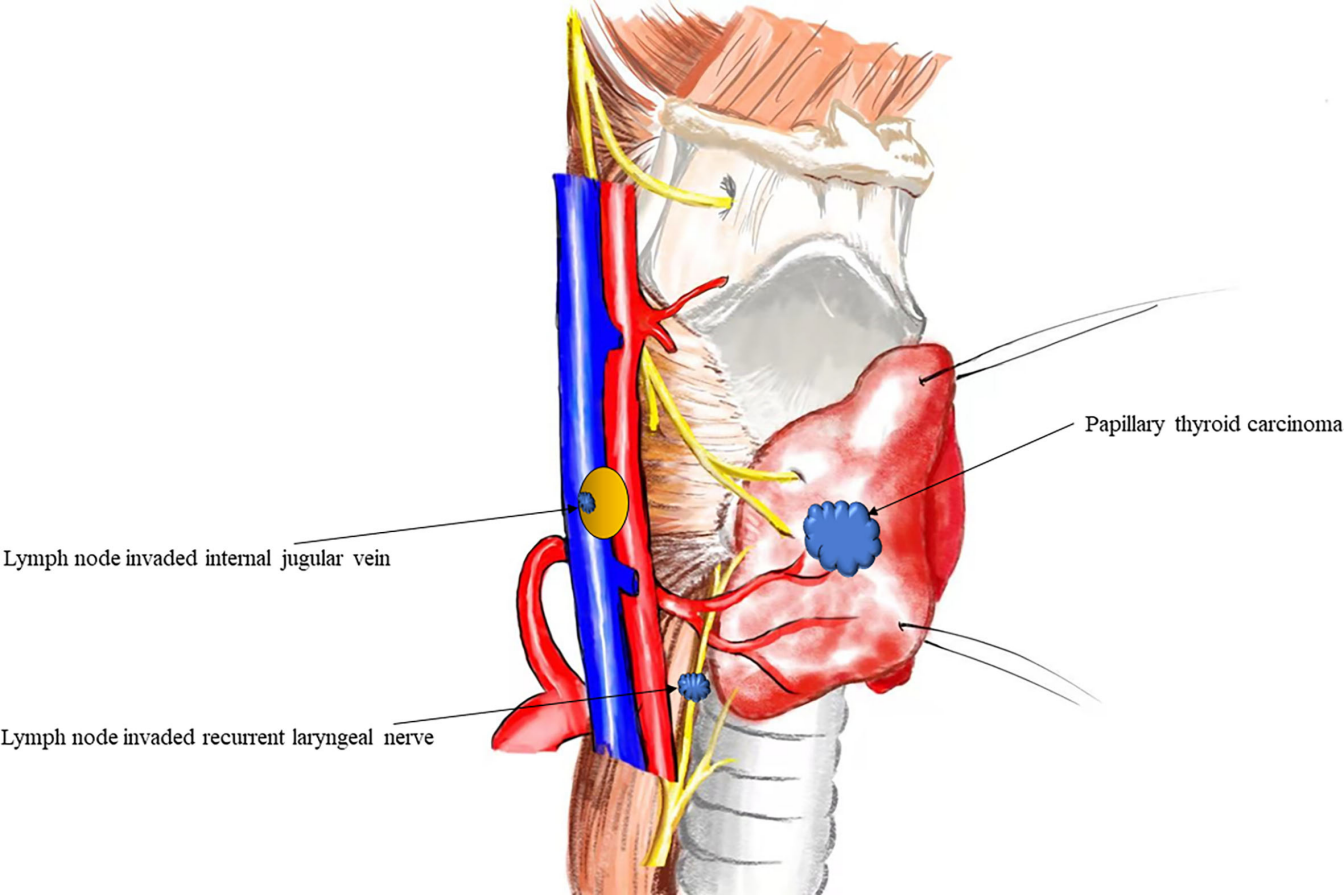
Anatomical diagram of extranodal extension in papillary thyroid cancer.

All clinical data were available through the electronic medical records, and patients with macroscopic ENE and macroscopic extrathyroidal extension were included in this study. ENE was defined as follows: intraoperative findings indicating that the organ had gross invasion by lymph node metastasis. As showed in **Figure 2** Extrathyroidal extension was defined as invasion of the larynx, trachea, esophagus, recurrent laryngeal nerve, mediastinal vessels, or carotid artery from the thyroid primary tumor site but not invasion of the sternothyroid or sternohyoid muscle (8, 9).

### Follow-Up Strategy for Papillary Thyroid Carcinoma Patients

During follow-up, the endpoint was recurrence-free survival (RFS) (without regional recurrence after surgery). All patients were regularly followed up by clinical evaluation, serum thyroglobulin level measurement, and ultrasound examinations 6 months after surgery. Recurrence was defined as structural recurrence and pathologically confirmed by fine-needle aspiration biopsy (FNAB) or distant recurrence. If the patient was lost to follow-up, their data were censored.

### Treatment Strategy for Papillary Thyroid Carcinoma Patients

All patients underwent open surgery. Thyroidectomy and cervical lymph node dissection were performed concurrently. Central lymph nodes were routinely dissected. Lateral cervical dissection was performed in patients with evidence of lateral cervical lymph node metastases through FNAB or preoperative imaging evidence. Important structures, such as the internal jugular arteriovenous, sternocleidomastoid muscle, and spinal accessory nerve, were spared. The therapeutic strategy for PTC with ENE was no residual resection or iodine 131 treatment. All PTC patients continued thyroid-stimulating hormone (TSH) suppression therapy after surgical treatment.

### Statistical Analysis

Statistical analysis was performed with R Studio (Version 1.3.1093). Normally distributed data are expressed as the mean ± standard deviation (SD), and skewed data are expressed as the interquartile range. Univariate and multivariable logistic regression analyses were used to compare two groups. Odds ratios (ORs) with 95% confidence intervals (CIs) were calculated. The log-rank test and Kaplan–Meier (K-M) curves were used for prognosis comparisons. Cox regression was performed to calculate the hazard ratios (HRs) and 95% CIs to determine the effects of ENE on RFS in PTC. *P* < 0.05 was considered statistically significant.

PSM analysis was conducted to minimize the effects of potential confounders on selection bias. PSM was performed with 1:1 matching and a caliper value of 0.02.

## Results

During the study period, 743 PTC patients were included. Among them, 220 were male and 523 were female. The age range of the patients was 18–76 years, with a mean of 42.84 years and an SD of 12.75 years. The baseline features of the PTC patients are presented in **Table 1**.

**Table 1:** Baseline characteristics of the PTC patients.

Variate	ENE (n = 34)	Non-ENE (n = 709)	*P*-value
Sex			
Female	21	502	0.259
Male	13	207	
Age (years)	47.94 ± 14.22	42.54 ± 12.59	
<55 years	23	575	0.015
≥55 years	11	134	
Size	15.26 ± 10.84	10.51 ± 8.16	
≤1 cm	12	466	0.001
>1 cm	22	243	
Multifocality			
Unifocal	20	526	0.047
Multifocal	14	175	
Extrathyroidal extension			
No	16	589	<0.001
Yes	18	120	
Hashimoto’s thyroiditis			
No	3	606	0.353
Yes	31	103	
Nodal stage			
N1a	3	500	<0.001
N1b	31	209	
Distant metastasis			
Yes	2	0	<0.001
No	32	709	
Complications			
No	32	693	0.179
Yes	2	16	

ENE, extranodal extension; PTC, papillary thyroid carcinoma.

### Clinical Characteristics of Extranodal Extension Patients

Thirty-four patients (34/743, 4.58%) with ENE were enrolled. The invaded organs were classified according to their location in the central neck compartment or lateral neck compartment. Thirty patients showed ENE of the central lymph nodes, and the median tumor size was 17.76 ± 13.66 mm. Lymph nodes mainly invaded the recurrent laryngeal nerve, followed by the surrounding adipose tissue and parathyroid gland. Twenty-two patients showed ENE of the lateral lymph nodes, and the median tumor size was 15.27 ± 10.87 mm. The internal jugular vein was the most frequently invaded of the cervical artery, followed by the adipose tissue, accessory nerve, and common carotid artery (**Table 2**).

**Table 2:** Extranodal extension in the central and lateral neck compartments.

Central neck compartment		Lateral neck compartment	
Tumor size	17.76 ± 13.66 mm	Tumor size	15.27 ± 10.87 mm
Recurrent laryngeal nerve	13	Internal jugular vein	13
Adipose tissue	4	Adipose tissue	9
Parathyroid gland	1	Accessory nerve	2
		Arteria carotis communis	1

### Risk Factors of Papillary Thyroid Carcinoma Patients With Extranodal Extension

Univariate analysis showed that age, tumor size, extrathyroidal extension, and nodal stage were associated with ENE, whereas sex, multifocality, and Hashimoto’s thyroiditis were not. We conducted multivariate analysis to further verify whether risk factors affected PTC patients with ENE. Our results showed that age, extrathyroidal extension, and nodal stage remained significantly different, but tumor size did not (**Table 3**).

**Table 3:** Logistic regression analysis of ENE-related risk factors.

Variate	Univariate analysis			Multivariate analysis		
	OR	95% CI	*P*-value	OR	95% CI	*P*-value
Sex	1.501	0.738~3.055	0.262	1.388	0.628~3.070	0.418
Age	1.033	1.006~1.062	0.017	1.036	1.006~1.066	0.016
Multifocality	2.012	0.996~4.065	0.051	11.894	0.875~4.100	0.105
Hashimoto’s thyroiditis	0.569	0.194~2.191	0.359	0.854	0.244~2.990	0.805
Tumor size	1.045	1.012~1.079	0.006	1.031	0.995~1.069	0.090
Extrathyroidal extension	5.522	2.738~11.136	<0.001	2.958	1.399~6.256	0.005
Nodal stage	24.721	7.475~81.750	<0.001	22.696	6.748~76.336	<0.001

ENE, extranodal extension; OR, odds ratio.

### Comparison of the Effect of Extranodal Extension on Patient Recurrence-Free Survival

**Figure 3:**
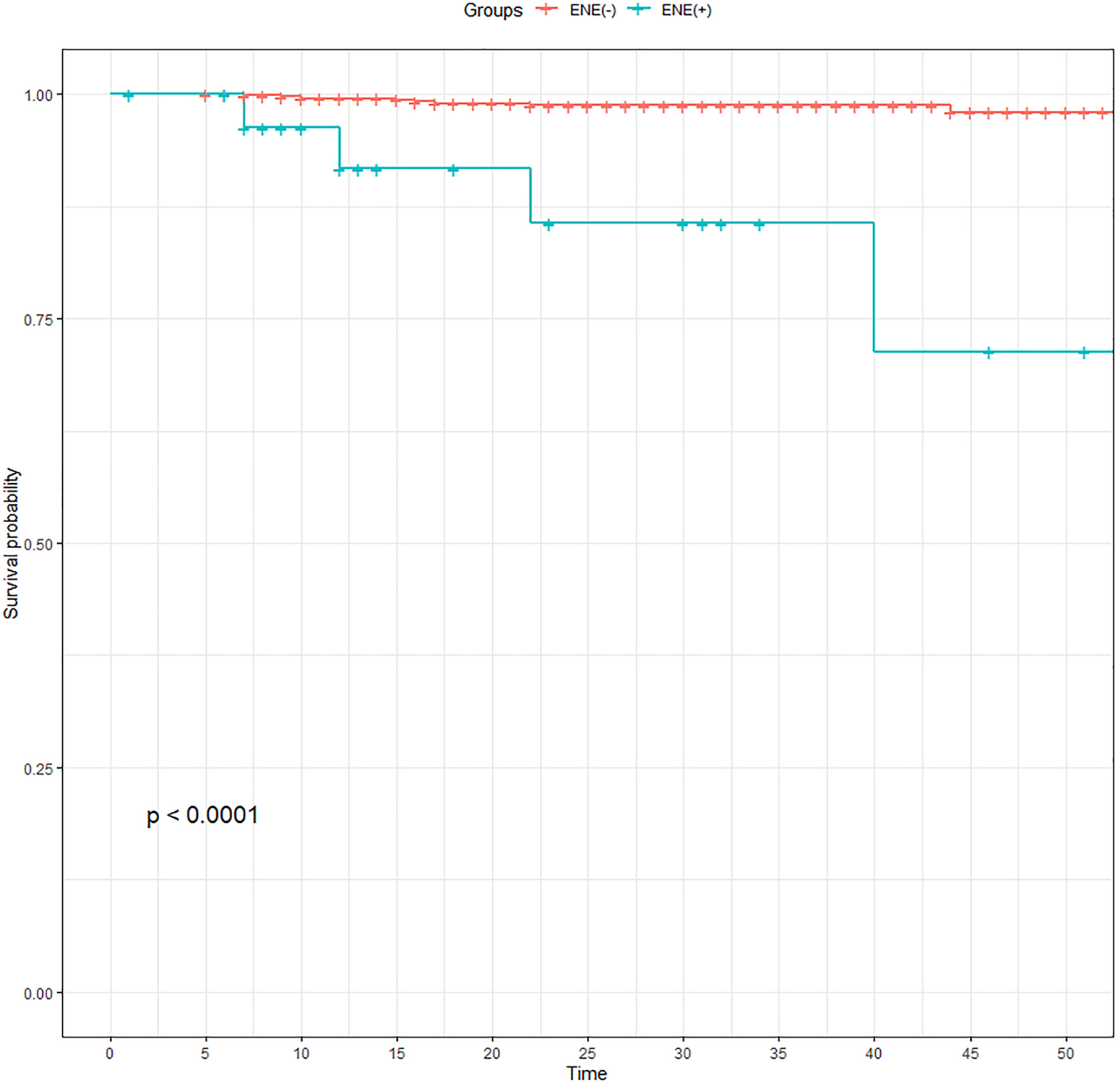
Comparison of recurrence-free survival of PTC patients before propensity score matching. ENE, extranodal extension; PTC, papillary thyroid carcinoma.

Twenty-three patients were lost to follow-up during the study period. The median follow-up time was 40.46 ± 15.51 months, and 15 of 720 PTC patients in this study experienced relapse. A comparison of the prognosis of the patients in the two groups indicated that the RFS of patients with ENE was lower than that of patients without ENE, and there was a significant difference between the two groups (*P <* 0.001; **Figure 3**).

**Figure 4:**
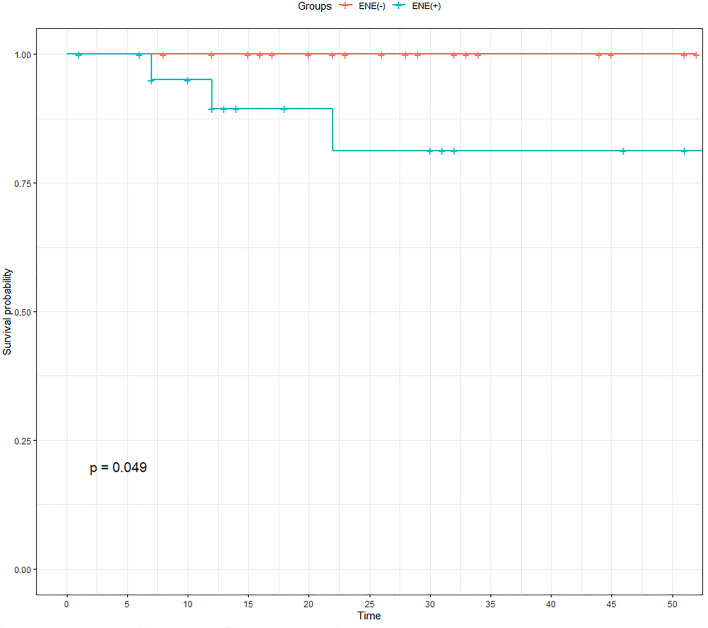
Comparison of recurrence-free survival of PTC patients after propensity score matching. ENE, extranodal extension; PTC, papillary thyroid carcinoma.

Then, we conducted PSM analysis to minimize the effects of potential confounders on selection bias. The model was adjusted for the following variables: sex, age, multifocality, tumor size, extrathyroidal extension, Hashimoto’s thyroiditis, and nodal stage. Finally, a total of 52 patients (26 one-to-one matched patients from each cohort) were compared, and RFS was subsequently compared (**Table 4**). The K-M curve indicated a statistically significant difference between the two groups after PSM (*P* = 0.049; **Figure 4**).

**Table 4:** Baseline clinical characteristics and procedure characteristics after PSM.

Variate	ENE (n = 26)	Non-ENE (n = 26)	*P*-value
Sex			
Female	18	20	0.532
Male	8	6	
Age (years)	44.08 ± 13.23	47.92 ± 13.40	
<55 years	20	14	0.303
≥55 years	6	12	
Size	15.69 ± 11.74	14.92 ± 8.01	
≤1 cm	6	8	0.784
>1 cm	20	18	
Multifocality			
Unifocal	15	15	1.000
Multifocal	11	11	
Extrathyroidal extension			
Yes	14	17	0.397
No	12	9	
Hashimoto’s thyroiditis			
Yes	2	6	0.124
No	24	20	
Nodal stage			
N1a	3	3	1.000
N1b	23	23	

ENE, extranodal extension; PSM, propensity score matching.

We further conducted Cox regression analysis to determine the effects of ENE on RFS in PTC. Our results showed that age, tumor size, and ENE were independent risk factors for RFS before PSM. Then, we balanced the confounding factors, and tumor size and ENE still showed statistical significance after PSM (**Table 5**).

**Table 5:** Cox regression analysis of patients before and after PSM.

Variate	Before PSM		After PSM	
	HR (95% CI)	*P*-value	HR (95% CI)	*P*-value
Age	1.041 (1.010~1.073)	0.009	1.060 (0.973~1.154)	0.182
Size	1.040 (1.005~1.076)	0.026	1.082 (1.029~1.139)	0.002
ENE	3.600 (1.378~9.405)	0.009	7.178 (1.191~43.249)	0.031

ENE, extranodal extension; HR, hazard ratio; PSM, propensity score matching.

## Discussion

In recent years, thyroid carcinoma has exhibited the most rapid increase in incidence among solid tumors, and PTC is the most common malignant thyroid cancer (10). Previous studies have shown that lymph node metastasis is very common, with an occurrence rate of 20%~80% in PTC. In our study, the lymph node metastasis rate was 35.48% (743/2094), which is consistent with that in the literature (3, 4). The American Thyroid Association risk stratification system includes the size and number of positive lymph nodes but not ENE (9). The presence of ENE is an important prognostic indicator in breast cancer and oral cancer. It has been previously suggested that a new risk stratification system incorporating ENE for predicting structural recurrent disease should be developed, but Shen et al. questioned this (11, 12). The prognostic role of ENE in PTC has long been controversial.

Previous studies, including a meta-analysis, suggest that the presence of ENE is a significant poor prognostic factor in PTC (13). Wu et al. (7) found that age ≥45 years and ENE are significant adverse independent prognostic factors for patients with PTC. Suh et al. (13) showed that PTC patients with ENE had a 2.01-fold higher risk of recurrence and a 3.37-fold higher risk of death than patients without ENE. At the same time, our results show that ENE is an independent risk factor for RFS. After PSM, we further confirmed the relationship between ENE and prognosis. Therefore, ENE has been suggested as a potential prognostic factor in previous studies.

The relationship between ENE and prognosis was further analyzed using PSM. PSM is an effective statistical method that can process nonrandomized study data (such as RWD) and effectively reduce the confounding factors to fully equalize differences between the study groups (14). For PSM, we adjusted for confounding factors that may have an impact on prognosis. Indeed, the independent prognostic predictive capability of ENE was retained even after adjustment for confounding factors.

This study also showed a significant association between ENE and the clinicopathological features of PTC, such as age, tumor size, extrathyroidal extension, and nodal stage. Roh et al. (15) found that age, tumor size, and nodal stage were significant factors for predicting ENE, which is similar to our research results. However, our study showed that tumor size was the only meaningful factor in the univariate analysis, and its predictive advantage was not seen in the multivariate analysis; it will be the subject of further study. Jointly, the two studies suggest that ENE is more likely to occur in older PTC patients with lymph node metastases in the lateral neck compartment. At the same time, various studies have confirmed the significant correlation between extrathyroidal extension and ENE. Kim et al. (16) reported that extrathyroidal extension was an independent predictor of ENE in a retrospective analysis of 1,693 PTC patients. In a study of 193 PTC patients with cervical lymph node metastasis, Clain et al. (17) found that the incidence of ENE increased 13-fold when patients with cervical lymph node metastasis also had extrathyroidal extension, which is consistent with our research results. Therefore, patients with these two clinical factors should be aware of the increased potential for ENE and the possible prognostic implications.

In the central neck compartment, the recurrent laryngeal nerve was most frequently infiltrated by lymph nodes with ENE. Moritani (8) found that there was a lower probability of neural palsy with ENE than with tumors directly invading the recurrent laryngeal nerve, so PTC patients often have no obvious clinical manifestations, such as hoarseness, before surgery. Ultrasound has certain significance for swollen lymph nodes with fusion signs, microcalcifications, cystic degeneration, and a ratio of length to short diameter of <2 to demonstrate ENE (18), but Alpert et al. (19) pointed out that ENE is not positively correlated with lymph node size, with lymph nodes less than 1 cm in diameter also being prone to ENE. The sensitivity of detecting lymph nodes invading nerves through ultrasound or neck enhanced computed tomography (CT) and other imaging data is low, so it is difficult to identify ENE with nerve involvement before surgery.

There are still some shortcomings in this study. 1) The follow-up time of this study is brief, and we will continue to follow-up this group of patients to further compare the difference in the long-term survival of patients. 2) This study focused on the prognostic significance of patients with macroscopic ENE. Further comprehensive studies on the prognostic significance of microscopic ENE are expected.

In summary, ENE is relatively uncommon in thyroid carcinoma, accounting for only 4.58% of all cases of lymph node metastasis in this study. Age ≥55 years, extrathyroidal extension, and lateral cervical lymph node metastasis have been identified as independent risk factors for ENE. ENE is an independent prognostic factor in PTC.

## Data Availability Statement

The raw data supporting the conclusions of this article will be made available by the authors without undue reservation.

## Ethics Statement

The studies involving human participants were reviewed and approved by Hangzhou First People’s Hospital. The patients/participants provided their written informed consent to participate in this study.

## Author Contributions

D-CL and T-HZ: drafted the work, designed the work, collected and analyzed data, revised the article for important content, final approval of the article to be published, agreement to be accountable for the work. FW: drafted the work, designed the work, collected and analyzed data, wrote the article, revised the article for important content, final approval of the article to be published, agreement to be accountable for the work. K-NL: designed the work, collected and analyzed data, revised the article for important content, final approval of the article to be published, and agreement to be accountable for the work. L-LM: collected and analyzed data, revised the article for important content, final approval of the article to be published, and agreement to be accountable for the work. L-QZ: collected and analyzed data, final approval of the article to be published, and agreement to be accountable for the work. K-CJ: collected and analyzed data, final approval of the article to be published, and agreement to be accountable for the work. YZ: drafted the work, designed the work, revised the article for important content, final approval of the article to be published, and agreement to be accountable for the work. BL and W-JZ: Statistical analysis and data processing, final approval of the article to be published, and agreement to be accountable for the work. All authors contributed to the article and approved the submitted version.

## Funding

This work was supported by the Medical Health Science and Technology Project of Zhejiang Provincial Health Commission (Grant numbers 0020190490 and 0020190151), the Medical and Health Technology Project of Hangzhou (Grant number A20200432), and the Zhejiang Provincial Medical and Health Technology Project (Grant number 2019RC240).

## Conflict of Interest

The authors declare that the research was conducted in the absence of any commercial or financial relationships that could be construed as a potential conflict of interest.

## Publisher’s Note

All claims expressed in this article are solely those of the authors and do not necessarily represent those of their affiliated organizations, or those of the publisher, the editors and the reviewers. Any product that may be evaluated in this article, or claim that may be made by its manufacturer, is not guaranteed or endorsed by the publisher.
